# The association between helicobacter pylori infection and erosive gastroesophageal reflux disease; a cross-sectional study

**DOI:** 10.1186/s12879-022-07278-6

**Published:** 2022-03-19

**Authors:** Ramin Niknam, Kamran Bagheri Lankarani, Mohsen Moghadami, Seyed Alireza Taghavi, Leila Zahiri, Mohammad Javad Fallahi

**Affiliations:** 1 Gastroenterohepatology Research Center, Shiraz University of Medical Sciences, Shiraz, Iran; 2 Health Policy Research Center, Shiraz University of Medical Sciences, Shiraz, Iran; 3 Clinical Microbiology Research Center, Shiraz University of Medical Sciences, Shiraz, Iran; 4 Thoracic and Vascular Surgery Research Center, Shiraz University of Medical Sciences, Shiraz, Iran

**Keywords:** Helicobacter pylori, Gastroesophageal reflux disease, Esophagus, Endoscopic findings

## Abstract

**Background:**

The association between H. pylori (Helicobacter pylori) infection and gastroesophageal reflux disease (GERD) is a complex and confusing subject. The aim of this study was to evaluate the association between helicobacter pylori infection and erosive gastroesophageal reflux disease.

**Method:**

In a cross-sectional study, all patients referred for endoscopy due to dyspepsia were enrolled. The diagnosis of erosive GERD was made by endoscopy. Patients with normal esophagus were selected as comparison group. Random gastric biopsies were taken from all participants to diagnose H. pylori infection.

**Result:**

In total, 1916 patients were included in this study, of whom 45.6% had GERD. The mean age (SD) was 42.95 (16.32). Overall, 1442 (75.3%) patients were positive for H. pylori infection. The frequency of H. pylori infection in mild GERD patients was higher than the severe GERD, but this difference was not significant (P = 0.214). Except for sociodemographic status (P < 0.001), other variables including gender, age, ethnicity, body mass index (BMI), smoking, and presence of hiatus hernia in patients had no significant association with the frequency of H. pylori infection. According to Robust Poisson regression models analysis, the association of H. pylori (PR 1.026; 95% CI 0.990–1.064; P = 0.158) and sociodemographic status were not significantly different between the two groups. But smoking, increased BMI, older age, presence of hiatus hernia, and peptic ulcer diseases were significantly associated with GERD compared with the non-GERD group.

**Conclusion:**

In our results, there was no association between H. pylori infection and erosive GERD. Further studies are recommended.

## Background

Gastroesophageal reflux disease (GERD), as a common gastrointestinal (GI) disorder, refers to symptoms or tissue damage caused by retrograde movement of the stomach contents to the esophagus. One of the most common complications of GERD is esophageal inflammation. Factors that may be contributing to the disease include lower esophageal sphincter dysfunction, increased numbers of transient lower esophageal sphincter relaxations, hiatus hernia, delayed gastric emptying, ineffective esophageal clearance, and the presence of an acid pocket [1–3].

Helicobacter pylori (H. pylori), as a gram-negative bacterium, plays an important role in the pathogenesis of different GI diseases including gastric ulcer, gastric mucosal lymphoma and gastric cancer [4]. However, the relationship between H. pylori and GERD is a complex and confusing subject that needs further investigation [5, 6]. There are several invasive and non-invasive diagnostic methods for diagnosing H. pylori infection [7].

Some studies have reported a higher prevalence of H. pylori in patients with GERD, while some have observed an inverse relationship. This relationship is difficult to justify because GERD is a disease that is affected by different risk factors include BMI, smoking, lifestyle habits, host factors and more [1–3].

To date, the true relationship of H. pylori to GERD is still unclear [4], and recent studies have shown that more research is needed to clarify this association, with a more focus on confounding factors in GERD and H. pylori [5, 6, 8]. The aim of this study was to evaluate the association between helicobacter pylori infection and erosive gastroesophageal reflux disease.

## Methods

### Population and study design

**Fig. 1:**
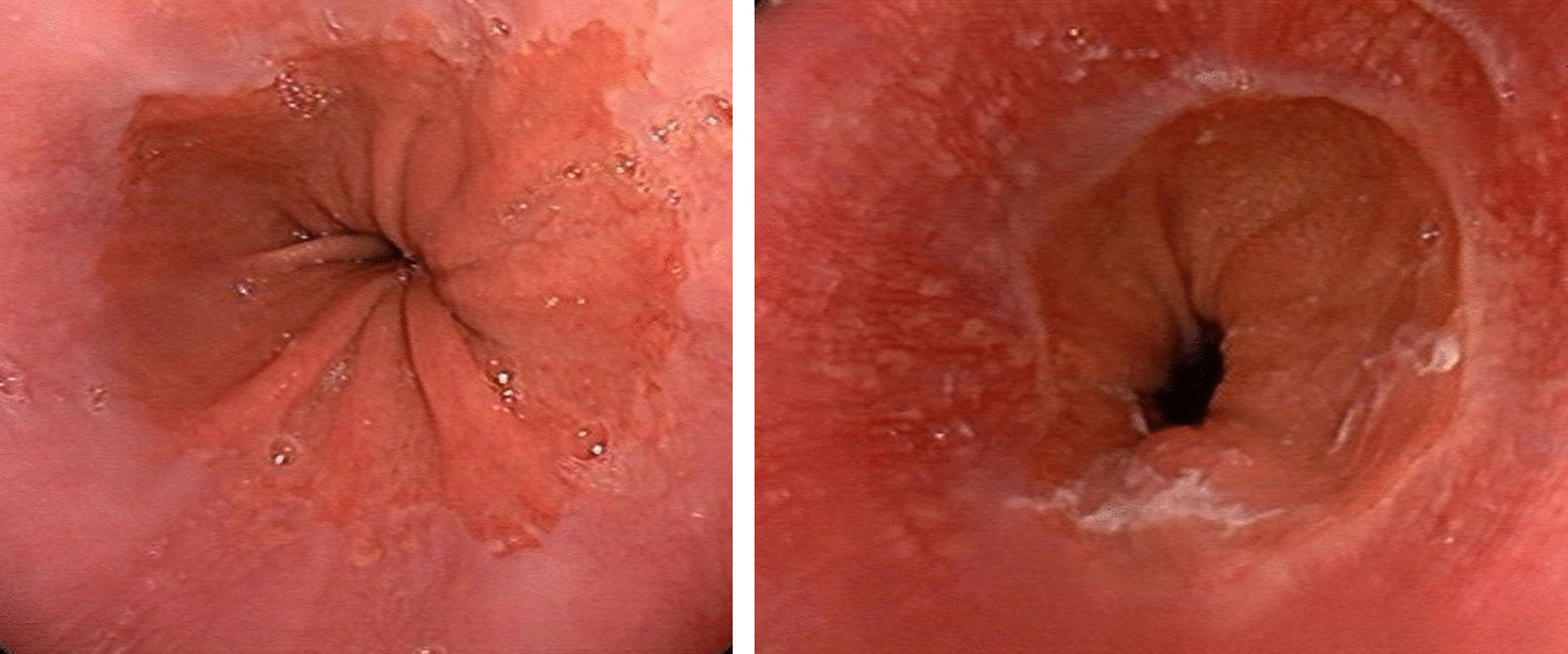
Endoscopic pictures of lower esophagus with (left) and without (right) erosive esophagitis

In this cross-sectional study, we evaluated the frequency of H. pylori infection in cases with endoscopic diagnosis of erosive GERD from March 2013 to November 2020 in Fars province, southern Iran. For this purpose, all consecutive Iranian patients referred to the endoscopy unit for esophagogastroduodenoscopy (EGD) due to dyspepsia were evaluated for erosive GERD. Diagnosis of dyspepsia was based on one or more clinical findings including epigastric pain, postprandial fullness, early satiation, epigastric burning, bloating in the upper abdomen, heartburn, nausea, and belching. Participants with abnormal esophageal endoscopy other than GERD were excluded. In order to compare the GERD group with a comparison group, we selected other participants from the same referred dyspepsia patients whose esophageal mucosa was completely normal at endoscopy (Fig. 1). Patients with the following conditions were excluded in both groups: history of H. pylori eradication, recent treatment with H2 blocker or proton pump inhibitors or non-steroidal anti-inflammatory drugs (NSAIDs) or medications induced GERD (e.g. anticholinergics, inhaled bronchodilators, and birth control pills), esophageal or gastric surgery, upper GI malignancy, and participants with poor cooperation. A checklist of EGD findings was filled out by gastroenterologist including of esophagus, stomach, and duodenum. An interviewer who was trained before starting the study, collected and recorded different demographic variables including age, sex, height, weight, sociodemographic status, smoking, and also histological reports of H. pylori in the checklist. Finally, the GERD group was compared with the non-GERD group in terms of H. pylori, considering the effect of confounding factors.

### Esophagogastroduodenoscopy

All upper EGD were performed by a gastroenterologist to determine the presence or absence of erosive GERD and to evaluate its severity (Fig. 1). In order to fully evaluate the upper GI tract, gastric and duodenal endoscopic findings were also examined. Gastric and duodenal endoscopic findings were divided into three groups: ulcerative, abnormal non-ulcerative (any evidence of mucosal lesion without ulcer), and normal [9]. Hiatus hernia was diagnosed when the apparent separation between squamocolumnar junction and diaphragmatic impression was greater than two centimeter during quiet respiration [10].

**Fig. 2:**
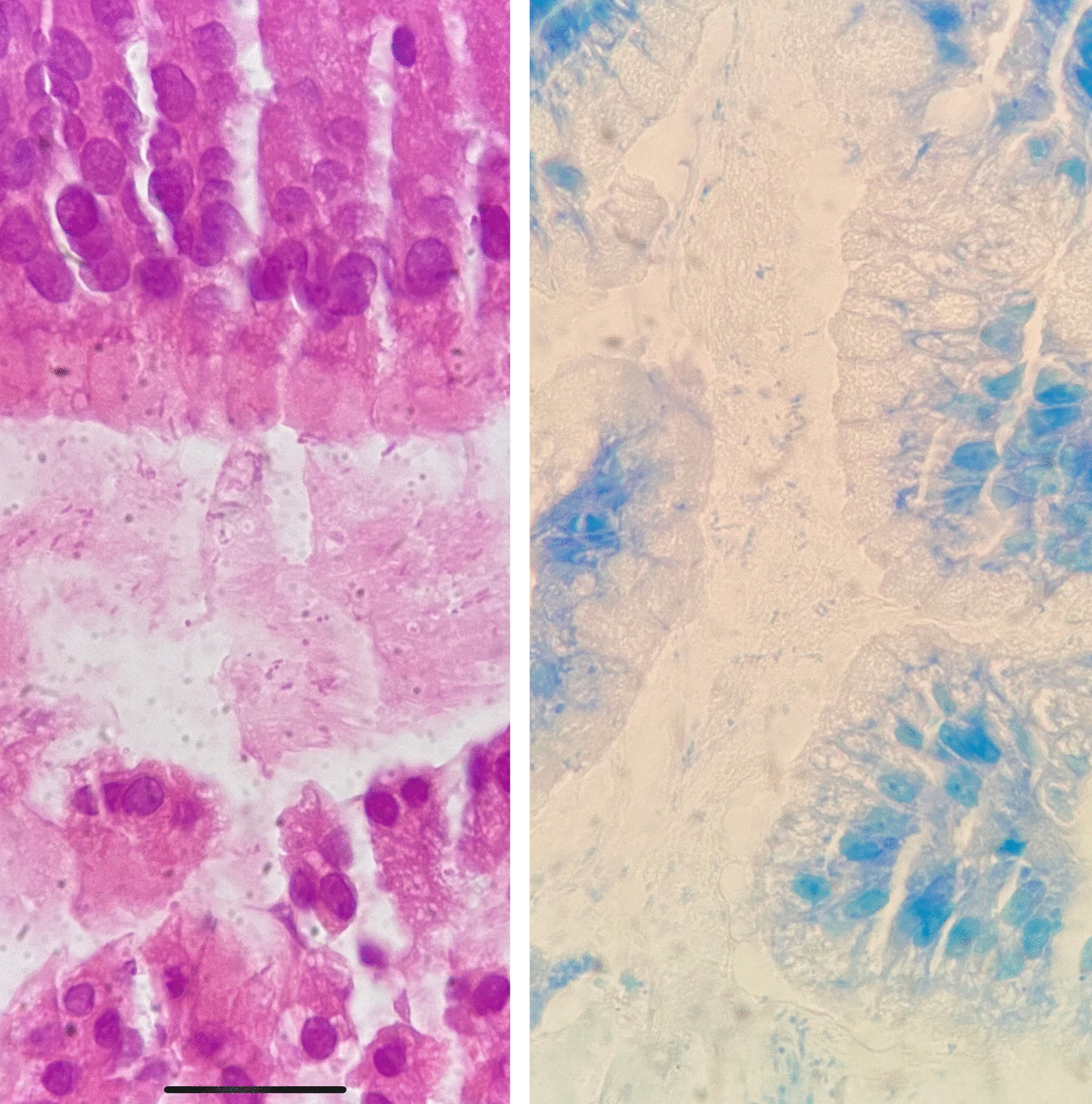
Section of gastric biopsies that showed Helicobacter pylori in hematoxylin and eosin staining (left) and Giemsa staining (right)

To detect H. pylori infection, random biopsies from the antrum and body of stomach were obtained from all participants in the GERD and non-GERD groups. The samples were transferred to the laboratory in 10% formalin and under appropriate conditions. For histological diagnosis of H. pylori, staining was performed with Hematoxylin and eosin and also Giemsa staining (Fig. 2).

### Diagnosis and definition of GERD severity

The diagnosis of erosive GERD was made by EGD and the Los Angeles classification was used to grade the esophagitis. One or more mucosal breaks confined to the mucosal folds (each no longer than 5 mm), was defined as grade A. At least 1 mucosal break greater than 5 mm long confined to the mucosal folds, was defined as grade B. At least 1 mucosal break continuous between the tops of 2 or more mucosal folds, was defined as grade C. Circumferential mucosal break, was defined as grade D [11, 12].

### Ethical approval/statement

This study was performed after obtaining the approval of Shiraz university ethical committee and Institutional Review Board (ID number: 93-01-13-8789) and based on Declaration of Helsinki regarding ethical principles for medical research. Written informed consent was obtained from all patients or their legal guardians to use their medical records in this study.

### Statistical analysis

Continuous data were calculated as means and standard deviations, whereas categorical parameters were expressed as percentages. Chi-squared test, T-test, and One-way ANOVA were used to capture the main differences between subjects, where appropriate. Robust Poisson regression analysis was used for estimating prevalence ratios (PRs) and confidence intervals (CIs) to evaluate the association of various independent variables on the GERD. For regression analysis, a cut off “PV < 0.3” in univariate analysis was used for inclusion in multivariate analysis. All analyses were performed with the commercial software “Statistical Package for the Social Sciences” (SPSS version 25.0, IBM, Chicago, USA). A P value of < 0.05 was considered statistically significant.

## Results

**Fig. 3:**
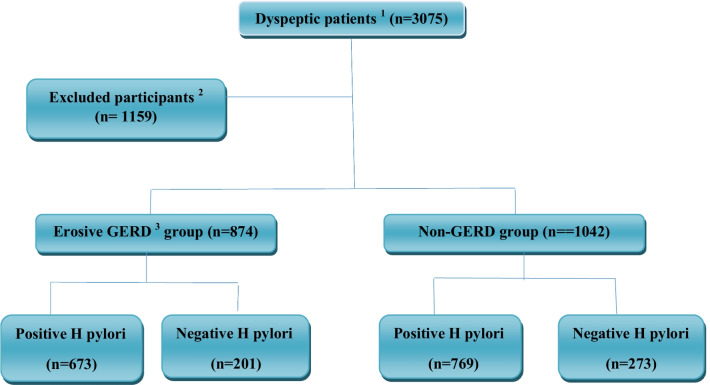
Flow diagram for the patient’s’ selection process. ^1^All consecutive patients referred to the endoscopy unit for esophagogastroduodenoscopy because of dyspepsia. ^2^Patients with the following conditions were excluded: history of H. pylori eradication, recent treatment with H2 blocker or proton pump inhibitors or NSAIDs or medications induced GERD (e.g. anticholinergics, selective serotonin reuptake inhibitor, inhaled bronchodilators, and birth control pills), esophageal or gastric surgery, upper gastrointestinal malignancy, and participants with poor cooperation. ^3^The diagnosis of erosive gastroesophageal reflux disease was made by esophagogastroduodenoscopy. GERD, gastroesophageal reflux disease

In total, 1916 patients were included in this study, of whom 874 (45.6%) had GERD and 1042 (54.4%) participants were considered as the non-GERD group (Fig. 3). The mean age (SD) was 42.95 (16.32) ranging from 19 to 90. 672 (35.1%) and 1244 (64.9%) patients were male and female, respectively. Mean age in males and females was 46.17 ± 18.15 and 41.22 ± 14.96, respectively. 1402 (73.2%) patients were rural residents and 183 (9.6%) were smokers. The demographic characteristics of the participants are shown in Table 1.

**Table 1:** Demographic characteristics of participants with dyspepsia (n = 1916)

Gender	
Male	672 (35.1%)
Female	1244 (64.9%)
Age distribution (yrs.)	
< 30	486 (25.4%)
30–39	438 (22.9%)
40–49	380 (19.8%)
50–59	293 (15.3%)
≥ 60	319 (16.6%)
Age (yrs.); Mean ± SD	42.95 ± 16.32
Provinces	
Fars	1460 (76.2%)
Lorestan	280 (14.6%)
West Azerbaijan/East Azerbaijan	108 (5.6%)
Others	68 (3.5%)
Sociodemographic status	
Rural	1402 (73.2%)
Urban age	514 (26.8%)
Body mass index (kg/m^2^); Mean ± SD	24.54 ± 5.48
Cigarette smoking	183 (9.6%)
Hiatus hernia	223 (11.6%)

Overall, 1442 (75.3%) patients were positive and the others were H. pylori negative. Except for sociodemographic status (P < 0.001), other variables including gender (P = 0.063), age (P = 0.695), ethnicity (P = 0.392), body mass index (BMI) (P = 0.236), smoking (P = 0.682), and presence of hiatus hernia (P = 0.601) had no significant association with the frequency of H. pylori infection (Table 2).

**Table 2:** Demographic characteristics of patients considering the presence of H. pylori and erosive gastroesophageal reflux disease (n = 1916)

Variable	Erosive gastroesophageal reflux disease			Helicobacter pylori infection		
	Yes	No	P value	Positive	Negative	P value
Gender^a^			0.36			0.06
Male	316 (36.2%)	356 (34.2%)		489 (33.9%)	183 (38.6%)	
Female	558 (63.8%)	686 (65.8%)		953 (66.1%)	291 (61.4%)	
Age (yrs.); Mean ± SD^b^	44.3 ± 16.7	41.8 ± 15.9	0.001	42.9 ± 15.9	43.2 ± 17.4	0.69
Sociodemographic status^a^			0.21			< 0.001
Rural	599 (68.5%)	686 (65.8%)		1055 (73.2%)	230 (48.5%)	
Urban	275 (31.5%)	356 (34.2%)		387 (26.8%)	244 (51.5%)	
Provinces^a^			0.43			0.39
Fars	651 (74.5%)	809 (77.6%)		1111 (77.0%)	349 (73.6%)	
Lorestan	139 (15.9%)	56 (13.5%)		207 (14.4%)	73 (15.4%)	
West Azerbaijan/East Azerbaijan	52(5.9%)	141 (5.4%)		76 (5.3%)	32 (6.8%)	
Others	32 (3.7%)	36 (3.5%)		48 (3.3%)	20 (4.2%)	
Body mass index (kg/m); Mean ± SD^b^	24.9 ± 5.44	24.3 ± 5.5	0.02	24.6 ± 5.5	24.3 ± 5.5	0.24
Cigarette smoking^a^	115 (13.2%)	68 (6.5%)	< 0.001	140 (9.7%)	43 (9.1%)	0.68
Hiatus hernia^a^	151 (17.3%)	72 (6.9%)	< 0.001	171 (11.9%)	52 (11.0%)	0.6

^a^Chi-square test

^b^T-test

Among patients with GERD, the presence of hiatus hernia, age, BMI, and smoking of patients in GERD group were significantly higher than non-GERD group (P < 0.05) but there was no significant difference in gender, sociodemographic status, and ethnicity between two groups (Table 2).

H. pylori infection was diagnosed in 673 (77.0%) patients in the GERD group, while 769 (73.8%) in the non-GERD group were positive for H. pylori infection. Frequency of H. pylori infection in the GERD group was higher than in the non-GERD group but there was no significant difference (P = 0.106). On the other hand, H. pylori infection was detected in 587 (77.6%) patients in mild GERD patients (LA grade A and B), while 39 (69.6%) in severe GERD (LA grade C and D) were positive for H. pylori infection. Although the frequency of H. pylori infection in mild GERD patients (LA grade A and B) was higher than the severe GERD (LA grade C and D), this difference was not significant (P = 0.214) (Table 3).

**Table 3:** Comparison of the frequency of H. pylori infection in dyspeptic patients considering the presence and grades of GERD (n = 1916)

Endoscopic findings	Positive H. pylori	Negative H. pylori	P value
Groups; N (%)^a^			0.106
GERD	673 (77.0%)	201 (23.0%)	
Non-GERD	769 (73.8%)	273 (26.2%)	
Grades of GERD; N (%)^a,b^			0.214
LA grade A	322 (75.8%)	103 (24.2%)	
LA grade B	265 (80.1%)	66 (19.9%)	
LA grade C	26 (66.7%)	13 (33.3%)	
LA grade D	13 (76.5%)	4 (23.5%)	

H. pylori, Helicobacter pylori; GERD, gastroesophageal reflux disease

^a^Chi-square test

^b^The Los Angeles Classification of gastroesophageal reflux disease was used for grading

Comparison of the frequency of H. pylori infection in patients with and without GERD in different types of gastroduodenal endoscopic findings is presented in Table 4. Among patients with GERD, the frequency of H. pylori infection was significantly higher in those with gastric abnormal lesions include ulcerative lesions than normal gastric findings (P = 0.006) but in non-GERD group, this difference was not significant (P = 0.068). On the other hand, the frequency of H. pylori infection in both GERD (P = 0.042) and non-GERD (P = 0.006) groups in duodenal ulcer patients was significantly higher than normal endoscopic findings (Table 4).

**Table 4:** Gastroduodenal endoscopic findings in GERD and non-GERD patients (n = 1916)

Endoscopic findings	GERD group	Non-GERD group	P value^a^
Gastric endoscopic findings; N (%)			0.030
Normal	492 (45.6%)	588 (54.4%)	
Ulcerative	23 (31.1%)	51 (68.9%)	
Abnormal non-ulcerative^b^	359 (47.1%)	403 (52.9%)	
Duodenal endoscopic findings; N (%)			0.015
Normal	760 (46.4%)	879 (53.6%)	
Ulcerative	53 (34.6%)	100 (65.4%)	
Abnormal non-ulcerative^b^	61 (49.2%)	63 (50.8%)	

*GERD* gastroesophageal reflux disease

^a^Chi-square test

^b^Included nodularity, erosion, erythema, and atrophic mucosa

The demographic and clinical characteristics and distribution of participants according to the BMI classification are shown in Table 5. Overall, the BMI (SD) was 24.54 (5.48) kg/m^2^, of which 1126 (58.8%) were in the normal weight group. Although 107 (5.6%) of patients were underweight, 480 (25.1%) and 203 (10.6%) were overweight and obese, respectively. There was a significant relationship between BMI and the presence of GERD (P = 0.001) as well as the sociological status (P = 0.007) and presence of hiatus hernia (P = < 0.001) of the participants. There was no significant difference between BMI classification and frequency of other variables such as gender (P = 0.134), age (P = 0.409), gastric findings (P = 0.073), duodenal findings (P = 0.629), cigarette smoking (P = 0.053), and H. pylori infection (P = 0.345).

**Table 5:** Distribution of demographic and clinical features of participants (n = 1916) according to BMI classification

Variables	Underweight; N (%) 107 (5.6%)	Normal; N (%) 1126 (58.8%)	Overweight; N (%) 480 (25.1%)	Obese; N (%) 203(10.6%)	P value
Gender^a^					0.134
Male	45 (42.1%)	388 (34.5%)	178 (37.1%)	61 (30.0%)	
Female	62 (57.9%)	738 (65.5%)	302 (62.9%)	142 (70.0%)	
Age (yrs.); Mean ± SD^b^	40.81 ± 16.35	43.36 ± 16.34	42.53 ± 16.51	42.82 ± 15.75	0.409
H. pylori infection^a^					0.345
Positive	77 (72.0%)	841 (74.7%)	375 (78.1%)	149 (73.4%)	
Negative	30 (28.0%)	285 (25.3%)	105 (21.9%)	54 (26.6%)	
Esophageal findings^a^					0.001
GERD	44 (41.1%)	477(42.4%)	245 (51.0%)	108 (53.2%)	
Non-GERD	63 (58.9%)	649 (57.6%)	235 (49.0%)	95 (46.8%)	
Gastric findings^a^					0.073
Normal	70 (65.4%)	615 (54.6%)	277 (57.7%)	118 (58.1%)	
Ulcerative	6 (5.6%)	42 (3.7%)	14 (2.9%)	12 (5.9%)	
Abnormal non-ulcerative	31 (29.0%)	469 (41.7%)	189 (39.4%)	73 (36.0%)	
Duodenal findings^a^					0.629
Normal	95 (88.8%)	954 (84.7%)	414 (86.3%)	176 (86.7%)	
Ulcerative	9 (8.4%)	92 (8.2%)	35 (7.3%)	17 (8.4%)	
Abnormal non-ulcerative	3 (2.8%)	80 (7.1%)	31 (6.5%)	10 (4.9%)	
Cigarette smoking^a^	18 (16.8%)	107 (9.5%)	39 (8.1%)	19 (9.4%)	0.053
Sociodemographic status^a^					0.007
Rural	83 (77.6%)	763 (67.8%)	320 (66.7%)	119 (58.6%)	
Urban	24 (22.4%)	363 (32.2%)	160 (33.3%)	84 (41.4%)	
Hiatus hernia^a^	9 (8.4%)	108 (9.6%)	69 (14.4%)	37 (18.2%)	< 0.001

The body mass index (kg/m^2^) was classified according to the World Health Organization (WHO) classification into 4 groups of less than 18.5 as under-weight range, 18.5 to < 25 as normal, 25.0 to < 30 as over-weight range, and 30.0 or higher as obese range

BMI, body mass index; H. pylori, Helicobacter pylori

^a^Test: Chi-squared test

^b^Test: One-way ANOVA

Robust Poisson regression models was used for estimating the PRs and 95% CIs to evaluate the association of various independent variables on the GERD (Table 6). H. pylori (PR 1.026; 95% CI 0.990–1.064; P = 0.158) and sociodemographic status (PR 1.030; 95% CI 0.998–1.063; P = 0.064) were not significantly associated with GERD group than non-GERD group. However, smoking (PR 1.139; 95% CI 1.089–1.192; P < 0.001), increased BMI (PR 1.060; 95% CI 1.027–1.093; P < 0.001), presence of hiatus hernia (PR 1.140; 95% CI 1.095–1.188; P < 0.001), and increased age (PR 1.002; 95% CI 1.001–1.003; P < 0.001) were significantly associated with GERD group compared to non-GERD group using regression analysis. In addition, the gastric ulcer (PR 0.875; 95% CI 0.809–0.947; P = 0.001) and duodenal ulcer (PR 0.911; 95% CI 0.862–0.963; P = 0.001) were also positively associated with presence of GERD.

**Table 6:** Robust Poisson regression models estimating prevalence ratio (PR) and 95% confidence interval (CI) to evaluate the association of various independent variables on the GERD

Variable	Crude model		Adjusted model	
	PR (95% CI)	P value	PR (95% CI)	P value
Cigarette smoking		< 0.001		< 0.001
Yes	1.132 (1.082–1.186)		1.139 (1.089–1.192)	
No	1.0		1.0	
Body mass index^a^		< 0.001		< 0.001
Overweight range and obese	1.066 (1.033–1.100)		1.060 (1.027–1.093)	
Normal and under-weight	1.0		1.0	
Hiatus hernia		< 0.001		< 0.001
Yes	1.175 (1.129–1.223)		1.140 (1.095–1.188)	
No	1.0		1.0	
Gastric ulcer		< 0.001		0.001
Yes	0.897 (0.826–0.973)		0.875 (0.809–0.947)	
No	1.0		1.0	
Duodenal ulcer		0.004		0.001
Yes	0.919 (0.867–0.974)		0.911 (0.862–0.963)	
No	1.0		1.0	
Age	1.002 (1.001–1.003)	0.001	1.002 (1.001–1.003)	< 0.001
Sociodemographic status		0.211		0.064
Rural	1.021 (0.988–1.055)		1.030 (0.998–1.063)	
Urban	1.0		1.0	
Helicobacter pylori		0.106		0.158
Positive	1.030 (0.994–1.068)		1.026 (0.990–1.064)	
Negative	1.0		1.0	

^a^The body mass index (kg/m^2^) was classified according into two groups of less than 25 as normal and under-weight, 25 or higher as overweight range and obese

## Discussion

Our study showed that the frequency of H. pylori infection in the erosive GERD and non-GERD groups was not significantly different. In addition, there was no significant difference in the frequency of H. pylori between mild and severe GERD (Table 3). According to Robust Poisson regression models analysis, some variables including smoking, increased BMI, older age, presence of hiatus hernia, and peptic ulcer diseases (but not H. pylori infection) were significantly associated with GERD compared with the non-GERD group (Table 6). These results, in agreement with some studies [13–15], support the hypothesis that there is no association between the frequency of H. pylori infection and GERD.

GERD is a common GI disorder with different risk factors including obesity, smoking, alcohol use, pregnancy, scleroderma, and some foods or medications [1–3]. Lifestyle modification is recommended as the first step in the treatment of GERD. Proton pump inhibitors are the mainstay of medical treatment for GERD if medication is needed, although a possible link between long-term use of these drugs and an increased risk of some side effects has been shown [1, 2, 16]. H. pylori, as a common infection, plays an important role in the pathogenesis of various benign and malignant gastroduodenal diseases including gastric ulcer, gastric mucosal lymphoma and gastric cancer [4], however, there is still no agreement on its role in GERD. According to some reports, an inverse relationship between H. pylori and GERD has been observed [5, 17–20], but some other studies have not shown this relationship [13–15].

A study in healthy young Japanese volunteers, conducted by Tanaka et al., aimed to determine the prevalence and risk factors of H. pylori and GERD and their interrelationship. In this study, similar to our results, H. pylori infection had no effect on the prevalence of GERD, but obesity was a risk factor for GERD. They also showed that gender was a risk factor for GERD, but the frequency of smoking or abdominal hernia was not significantly different between groups that was different from our results [13]. Mahdi et al. investigated the association between CagA + H. pylori and GERD and compared them with the healthy group. They concluded that the presence of H. pylori in patients with GERD was significantly increased compared to controls group [21].

In a research from Iran, 470 patients with dyspepsia and GERD were studied. The rate of H. pylori infection was 78.1%, which was almost similar to our results (Table 3) but the mean age of our patients was lower than their participants. They found no relationship between hiatus hernia and H. pylori, which was inconsistent with our results [22]. In another study from Iran, they did not find any association between H. pylori in patients with GERD compared to controls [23], which was consistent with our results.

Grand et al. conducted a study to examine 184 patients with reflux symptoms who underwent endoscopy with biopsy, esophageal pH-metry, and manometry. They showed that the role of H. pylori infection in the development of GERD as well as in the pathogenesis of esophageal reflux esophagus was not significant but hiatus hernia was significantly associated with the presence of reflux esophagus [24]. In a study by Gisbert et al., they used pH-metry and endoscopy to diagnose GERD. In their research, H. pylori infection was not associated with GERD based on both procedures [25]. Another study based on esophageal manometry, 24-h pH monitoring, and EGD showed that GERD features, such as abnormal esophageal acid, erosive esophagus, or Barrett’s esophagus, were not related to H. pylori [26], which is consistent with our results.

A prospective study of 146 patients with GERD, to determine the prevalence of H. pylori infection, found that there was no significant evidence for an important role in H. pylori infection in causing GERD and erosive esophagitis. In addition, although there was a significant relationship between hiatus hernia and reflux esophagitis, there was no significant correlation between HP and hiatus hernia, which was completely consistent with our results [27]. Two other prospective evaluations by O'Connor et al. and Pieramico et al. also did not support the significant association between H. pylori infection and GERD [28, 29].

A study of 2508 GERD populations by Mari et al. showed that H. pylori infection was observed in 299 (11.9%) patients. Patients with GERD and H. pylori in this study were significantly younger, smoked more, and had less severe esophagitis, which was not similar to our study results [20]. In Another study by Wang et al., in a non-erosive esophageal esophagus, showed that H. pylori infection was inversely associated with GERD, whereas male hiatus hernia were important factors associated with GERD [17]. Other than the effect of hiatus hernia, other results of this study were inconsistent with our study.

Two studies from Korea showed that H. pylori seropositivity is preventive [18] and absence of H. pylori and male gender were associated with reflux esophagitis [19], which is not consistent with our study. But in one of the mentioned studies, reflux esophagitis was significantly associated with hiatal hernia and BMI that was similar to our results [19]. A study by Yalaki et al., aimed at comparing and evaluating the relationship between GERD and H. pylori in adult patients with gastric localization of H. pylori infection and its historical features, the incidence of H. pylori has been shown to be significantly lower in patients with GERD than in the control group. This result is not consistent with the results of our study [5].

In a research from Indonesia, 104 patients with dyspepsia was analyzed to determine the prevalence of GERD and its risk factors. 53.8% of their patients had GERD that, similar to our results, smoking was significantly associated with GERD and most participants were classified as LA grade A. They also showed that higher economies increase the risk of GERD [30]. In our study, although the frequency of H. pylori in the rural was significantly higher than the urban participants, there was no significant difference between the two groups in terms of GERD (Tables 2, 6).

As shown in this Table 4, among our patients with GERD, the frequency of H. pylori infection was significantly higher in those with gastric ulcer than normal gastric findings but in non-GERD group, this difference was not significant. Peptic ulcer disease is commonly associated disease with GERD [2] and EGD plays an important role in the diagnosis and differentiation of benign and malignant GI diseases and its complications include peptic ulcer disease and GERD [9–12]. In a retrospective research by Jie et al., 953 peptic ulcer patients, 180 peptic ulcers and GERD patients, and 298 GERD patients were analyzed. They concluded that in patients with GERD, the prevalence of H. pylori infection in gastric ulcer patients was higher than without gastric ulcer [31], which was consistent with our results (Table 4). Moreover, gastric and duodenal ulcers, but not H. pylori infection, were significantly associated with GERD compared with the non-GERD group, according to Robust Poisson regression models analysis (Table 6).

Different treatment regimens have been suggested for H. pylori and some reports have been published on the effect of H. pylori eradication on GERD, however, their results have been inconsistent [7, 32–38]. Although some reports have shown an inverse association between H. pylori eradication with GERD development [34, 36, 37], others have shown no beneficial effect of H. pylori eradication on GERD [32, 33, 35]. Finally, there is no consensus on the hypothesis that eradicating H. pylori may cause or worsen GERD [4, 39].

The relationship between H. pylori and GERD is a complex and confusing issue due to the influence of various pathophysiological factors between them [5, 6]. One reason for the heterogeneity of the results of previous researches to find the true relationship between H. pylori and GERD may be that the design of many studies was only to find a simple relationship between them, whereas in the final analysis of many of these reports, the effect of confounding factors for this association have not been measured. For instance, The H. pylori infection may make people susceptible to GERD by increasing gastric acid secretion, either directly infecting the gastric-type columnar epithelium, or by the action of noxious substances secreted by the infection into refluxed gastric juice [40]. H. pylori seems to lead to much more complex changes in the gastric mucosa, including the modification of afferent neural signals and the secretion of specific gastric hormones. Ghrelin is a hormone that is mainly produced and released by the stomach with numerous functions. Ghrelin, in addition to enhancing gastric secretion, has a potent prokinetic function in the LES; this phenomenon, together with impaired vaginal control, may play a role in the association of H. pylori infection with the development of GERD. Therefore, ghrelin and vagal activity may be missing links that partly explain the relationship between GERD and H. pylori infection [41].

The strength of our study was to analyze the association between GERD and H. pylori infection, taking into account many confounding factors. Other strengths of our study were the size of the considerable sample size, the presence of the comparison group, and the appropriate diagnostic evaluation for all participants. Our research also had limitations. One important limitation was that we included only erosive GERD patients, so the results of this study may not be generalizable to patients with non-erosive reflux disease. On the other hand, in the non-GERD group, there may be a number of patients with non-erosive-GERD, so, a study is recommended to compare non-erosive GERD groups with erosive GERD groups. Another limitation was that the effects of some pathophysiological factors for both GERD and H. pylori were not measured in this study. Detection of Helicobacter pylori by staining alone was another limitation of this study. Endoscopic biopsies to detect H. pylori in our study were sent to the laboratory as a mixture of gastric body and antrum in one sample container. It is recommended that in future studies, biopsies of different areas of the stomach be sent to the laboratory in separate sample containers for more accurate evaluation, including other complementary methods such as molecular methods. Finally, this study was performed only in one center without a control group of the general population, so a multicenter case–control study is recommended.

## Conclusions

Although our results support the hypothesis that there is no association between the frequency of H. pylori infection and erosive GERD, the available data do not provide sufficient evidence to define the true relationship between them and this issue remains controversial. We recommend further studies in this area.

## Data Availability

The datasets generated and/or analyzed during the current study are not publicly available due our research center policy but are available from the corresponding author on reasonable request.

## Abbreviations

H. pylori: Helicobacter pylori
GERD: Gastroesophageal reflux disease
BMI: Body mass index
GI: Gastrointestinal
EGD: Esophagogastroduodenoscopy
NSAIDs: Non-steroidal anti-inflammatory drugs
